# Clinical Indicators and Imaging Characteristics of Blunt Traumatic Diaphragmatic Injury: A Retrospective Single-Center Study

**DOI:** 10.3390/jcm14186562

**Published:** 2025-09-18

**Authors:** Hoon Ryu, Chun Sung Byun, Sungyup Kim, Keum Seok Bae, Il Hwan Park, Jin Rok Oh, Chan Young Kang, Jun Gi Kim, Young Un Choi

**Affiliations:** 1 Department of Surgery, Yonsei University Wonju College of Medicine, Wonju 26426, Republic of Korea; kaljarbigs@yonsei.ac.kr (H.R.); sykimvs@yonsei.ac.kr (S.K.); bksgs@yonsei.ac.kr (K.S.B.); witsumbe@yonsei.ac.kr (J.G.K.); 2 Department of Thoracic and Cardiovascular Surgery, Yonsei University Wonju College of Medicine, Wonju 26426, Republic of Korea; csbyun@yonsei.ac.kr (C.S.B.); nicecs@yonsei.ac.kr (I.H.P.); 3 Trauma Center, Wonju Severance Christian Hospital, Wonju 26426, Republic of Korea; jroh@yonsei.ac.kr (J.R.O.); chlrhkcy@naver.com (C.Y.K.); 4 Department of Orthopedic Surgery, Yonsei University Wonju College of Medicine, Wonju 26426, Republic of Korea

**Keywords:** emergency department, psychiatric diagnoses, self-harm method, suicide attempts, trauma patients

## Abstract

**Background/Objectives:** Blunt trauma injury of the diaphragm is uncommon. Even after imaging examination, accurate diagnosis remains difficult. We sought to identify clinical factors that raise suspicion of such injuries, which can be applied during the initial evaluation of trauma patients. **Methods:** We retrospectively analyzed patients with blunt trauma who were diagnosed with diaphragmatic injury between January 2015 and July 2025. Demographic variables, clinical findings, operative records, and imaging findings were reviewed. **Results:** The most common mechanism of injury in patients with diaphragmatic injury was traffic accidents (64.2%), and 77.4% were identified as severe injuries with an Injury Severity Score (ISS) ≥ 16. Computed tomography (CT) scans of these patients frequently showed hemothorax, hemoperitoneum, and pneumothorax, but 49.1% of cases did not show diaphragmatic injury on preoperative imaging. In these patients, pneumothorax, lower rib fractures, and liver injury were more common. Notably, pneumothorax strongly suggested the possibility of diaphragmatic injury in patients where intrathoracic herniation was not clear. **Conclusions:** In patients with polytrauma and unstable vital signs, CT evaluation of torso injuries and careful interpretation are essential. Even when CT does not reveal diaphragmatic injury, suspicion should be elevated in cases with high ISS accompanied by pneumothorax, hemoperitoneum, hemothorax, lower rib fractures, or extremity injuries. If the injury mechanism further raises clinical suspicion, repeated physical examinations and imaging studies should be performed. When suspicion remains high, surgical intervention should be considered to confirm the diagnosis.

## 1. Introduction

Trauma-related diaphragmatic injury is relatively uncommon, occurring in less than 1% of trauma cases [1]. Blunt traumatic diaphragmatic injury might be diagnosed using clinical evaluation and various radiological modalities; however, most findings are indirect, resulting in a low early detection rate [2].

Currently, the most important imaging test for diagnosing diaphragmatic injury is computed tomography (CT). However, two problems arise when performing an imaging diagnosis of patients with traumatic diaphragmatic injury. First, CT scans are difficult to obtain when the patient is unstable due to multiple injuries. Second, even when the patient is stable and a CT scan is performed, the CT scan may not clearly show diaphragm injury, leading to a missed diagnosis. Observation of abdominal organ herniation into the thoracic cavity reliably indicates diaphragmatic injury [3,4]. However, in the absence of observed herniation, discerning diaphragmatic injuries based on imaging findings alone is difficult [5].

For these reasons, this study aimed to identify clinical conditions and imaging findings suggestive of diaphragmatic injury to reduce misdiagnosis during the initial treatment of trauma patients. We analyzed clinical findings, demographic characteristics, and injury mechanisms suggestive of diaphragmatic injury in patients with blunt trauma diagnosed with diaphragmatic injury postoperatively.

## 2. Methods

### 2.1. Patients

In this retrospective study, we reviewed and analyzed the medical records of patients with blunt trauma who were admitted to the emergency department of our institution between 1 January 2015, and 31 July 2025, in whom diaphragmatic injury was confirmed intraoperatively. This study was conducted in accordance with the Declaration of Helsinki and was approved by the Institutional Review Board of Yonsei University Wonju College of Medicine (IRB no. CR 325058). The need to obtain informed patient consent for data use was waived because of the retrospective nature of the study and the use of only de-identified data from medical records.

Inclusion criteria

1. Trauma patients admitted to the emergency room of this hospital with a confirmed Diaphragm Rupture or Diaphragm Injury code.
2. Patients with detailed medical records.
3. Patients who underwent surgery and had a diaphragm clearly described in the surgical record.

Exclusion criteria

1. Patients with radiologically confirmed diaphragmatic damage but who could not undergo surgery (Death within 24 h, Death on arrival).
2. Penetrating injury patients.
3. Patients with unstable vital signs who underwent surgery without preoperative imaging.

We included trauma patients admitted with the diagnosis code “diaphragmatic rupture” or “diaphragmatic injury” who had complete and detailed medical records and whose diaphragmatic injury was confirmed by surgery. We excluded patients with radiologically confirmed diaphragmatic injury but who were not eligible for surgery and therefore had no surgical findings (dead within 24 h or dead on arrival), patients with penetrating injuries, patients with hemoperitoneum who underwent laparotomy without CT because of unstable vital signs, and patients who therefore could not undergo preoperative imaging and therefore had no preoperatively confirmed diaphragmatic injury by imaging. Among the patients included in the above study, one was a 12-year-old child, and the other 52 were all adults aged 26 years or older.

**Figure 1:**
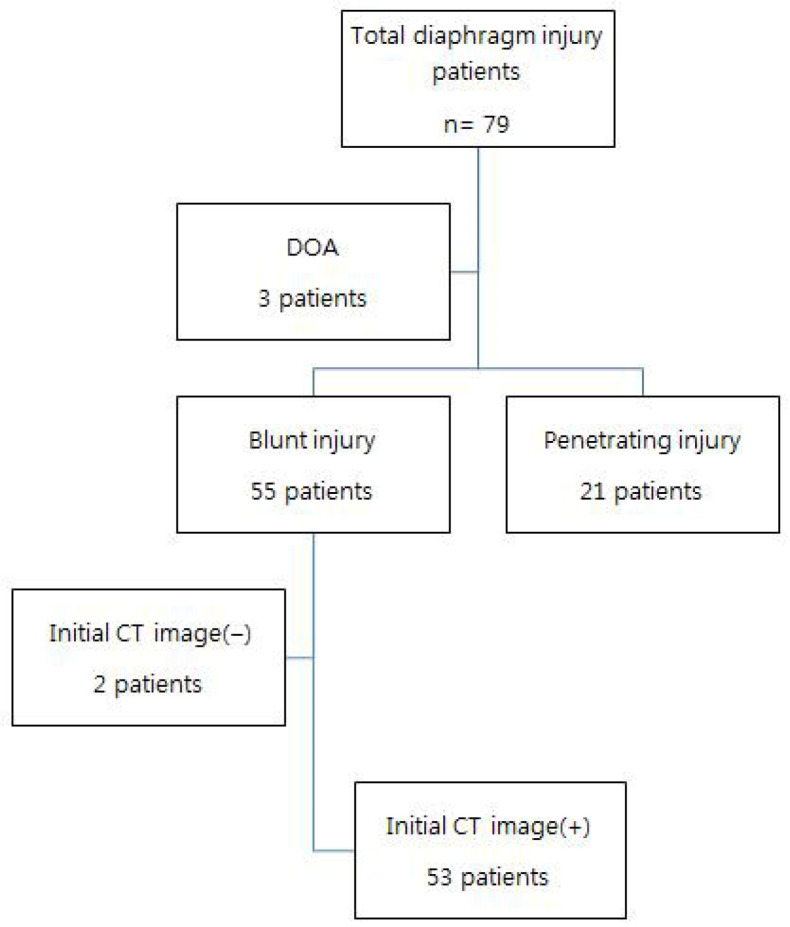
Flow-chart of inclusion of patients with blunt traumatic diaphragmatic injury. DOA, dead on arrival. (−), no imaging performed before surgery; (+), imaging performed before surgery.

Of the 79 patients with diaphragmatic injury who were admitted during the study period, 55 had blunt injuries, 21 had penetrating injuries, and 3 were dead on arrival. Among the patients with blunt injury, 2 had unstable vital signs and underwent surgery without imaging. Therefore, 53 patients were included in the final analysis (Figure 1).

### 2.2. Variables Analyzed

As demographic variables, we recorded sex and age. Clinical findings noted for analysis included maximum and minimum systolic blood pressure on arrival at the emergency department; time from arrival at thee emergency department to surgery; mechanism of injury (traffic accident [pedestrian, driver, passenger, or motorcyclist]; fall; other [slip, rolling, struck]); preoperative identification of diaphragmatic injury; Injury Severity Score (ISS); Abbreviated Injury Scale (AIS) scores for each body region; and intraoperative confirmation of the diaphragmatic injury site (left, right, or bilateral).

CT findings noted for analysis included abdominal organ herniation into the thoracic cavity, hemothorax, pneumothorax, fracture of the ipsilateral lower (6th–12th) ribs, liver laceration, splenic laceration, and bowel perforation. The imaging diagnoses of the above patients were initially confirmed by a thoracic trauma specialist, a surgical trauma specialist, and an emergency medicine trauma specialist upon transport to the emergency room and examination. The surgical trauma specialist then re-examined all images at the time of the study and reported the results regarding diaphragmatic injury.

### 2.3. Statistical Analysis

For categorical variables, data are presented as frequencies and percentages, and the chi-squared test was used for hypothesis testing. Fisher’s exact test was used when >20% of the cells in the contingency table had expected counts of <5 or when any cell had a frequency of 0.

For continuous variables, normality of data distribution was first tested using the Shapiro–Wilk test. Normally distributed variables are expressed as mean ± standard deviation and were analyzed using an independent samples *t*-test. Non-normally distributed variables are expressed as median and interquartile range and were analyzed using the Mann–Whitney U test.

Additionally, logistic regression analysis was performed to identify the factors predicting diaphragmatic injury in cases without abdominal organ herniation into the thoracic cavity. A stepwise variable selection method was used.

Statistical significance was defined as *p* < 0.05. All statistical analyses were performed using SAS version 9.4 (SAS Institute, Cary, NC, USA).

## 3. Results

### 3.1. General Demographics

The characteristics of the patients are summarized in Table 1. The average patient age was high at 56.4 years, and the most common mechanism of injury was traffic accidents, followed by falls. The left side was more prevalent than the right side, and it is noteworthy that 49.1% of cases had diaphragmatic injuries that were not confirmed radiologically before surgery. In addition to diaphragmatic injuries, other imaging findings were hemothorax, hemoperitoneum, and pneumothorax, in that order. Most patients suffered moderate to severe multiple trauma, and 77.4% had severe injuries with an ISS ≥ 16.

### 3.2. Comparison by Severity

Comparisons between severe and non-severe injury groups, defined by a threshold of ISS = 16, are summarized in Table 2. Severe injuries (ISS ≥ 16) were significantly more likely to have resulted from traffic accidents, as compared to non-severe cases (*p* < 0.0001), whereas falls and other injury mechanisms were more common in the non-severe group. Severe cases had significantly a shorter median time to surgery (*p* = 0.0003). The proportion of patients in whom surgery was delayed by >1 day was significantly smaller in the severe group (*p* = 0.0010).

Preoperative identification of diaphragmatic injury (*p* = 0.0410) and the presence of herniation (*p* = 0.0035) and hemoperitoneum (*p* = 0.0001) were significantly more common in the severe group. AIS scores ≥ 2 and ≥3 for the abdomen and extremities were also significantly more frequent among severe cases (abdomen AIS score ≥ 2: *p* = 0.0292; extremities AIS score ≥ 2, *p* < 0.0001; abdomen AIS score ≥ 3, *p* = 0.0256; extremities AIS score ≥ 3, *p* = 0.0256) (Table 2).

### 3.3. Comparison by Injury Site

All 52 patients, except one with bilateral injuries, were analyzed for their injury characteristics. Patients with left-sided injury are compared to those with right-sided injury in Table 3. Traffic accident-related injuries were significantly more common in the left-sided injury group, whereas falls were more frequent in the right-sided injury group (*p* = 0.0174). The left-sided injury group had a significantly shorter median time to surgery (*p* = 0.0277) and a higher preoperative diagnosis rate (*p* = 0.0021). No significant differences in AIS scores were found between the two groups.

### 3.4. Comparison by Preoperative Recognition

Characteristics of patients with and without a preoperative diagnosis of diaphragmatic injury are compared in Table 4. In patients diagnosed preoperatively, traffic accident-related injuries were significantly more frequent (*p* = 0.0290) and the median time to surgery was significantly shorter (*p* = 0.0434). A surgical delay >1 day was significantly more common in the undiagnosed group (*p* = 0.0156).

Anatomically, left-sided injuries were significantly more frequent in the preoperatively diagnosed group (*p* = 0.0033), and herniation was significantly more frequent in this group (*p* < 0.0001). In contrast, pneumothorax (*p* = 0.0075), rib fracture (*p* = 0.0214), and liver injury (*p* = 0.0243) were significantly more common in the undiagnosed group. No statistically significant differences in the AIS scores were observed between these groups.

### 3.5. Comparison by Presence of Abdominal Organ Herniation into the Thoracic Cavity

We next compared the characteristics of patients according to the presence or absence of herniation of abdominal organs into the thoracic cavity (Table 5). Patients with herniation were more likely to have sustained traffic accident-related injuries (*p* = 0.0321) and had a significantly shorter median time to surgery (*p* = 0.0249). Surgical delay of >1 day was significantly more common in the non-herniated than in the herniated group (*p* = 0.0156). Preoperative identification of diaphragmatic injury was also significantly more common in the herniated group (*p* < 0.0001).

Pneumothorax and rib fractures were more frequent in the non-herniated group (*p* < 0.0001 and *p* = 0.0145, respectively). Hemoperitoneum was more frequent in the herniated group (*p* = 0.0420), as was an AIS score ≥ 2 for injury to the extremities (*p* = 0.0169) (Table 5).

### 3.6. Analysis of Factors Predicting Diaphragmatic Injury Among Patients with Preoperatively Unrecognized Diaphragmatic Injury and in Non-Herniation Cases

Logistic regression was performed with “unrecognized preoperative diaphragmatic injury” as the event, and with all possible independent variables (sex, age, maximum/minimum systolic blood pressure, ISS, injury side, hemothorax, pneumothorax, ipsilateral lower rib fracture, hemoperitoneum, liver injury, splenic injury, mechanism of injury, and AIS score by body region) included using the stepwise selection method. The final model identified right-sided injury (odds ratio [OR] 10.832, 95% confidence interval [CI], 1.987–59.052) and herniation (OR 0.052, 95% CI: 0.010–0.280) as significant predictors.

For the event “absence of herniation,” logistic regression using the same variables as above identified pneumothorax (OR 53.759, 95% CI: 6.128–471.635) and AIS score ≥ 2 for extremity injury (OR 0.081, 95% CI: 0.009–0.707) as significant predictors (Table 6).

## 4. Discussion

### 4.1. Laterality of Diaphragmatic Injury in Blunt Trauma

Diaphragmatic injuries caused by blunt trauma occur predominantly on the left side and are frequently accompanied by injuries to other organs, which hampers early diagnosis in patients with unstable vital signs [6]. Anatomically, the left hemidiaphragm, particularly its medial, posterolateral, and tendinous portions, is congenitally weaker, whereas the right side is protected by the liver. Moreover, right-sided injuries are associated with high mortality rates, which increases the likelihood of being missed. Furthermore, while the cushioning effect of the liver may reduce injuries to the right hemidiaphragm, this effect also makes detection on imaging more difficult, resulting in a higher rate of missed diagnoses [7]. Some autopsy studies have reported that left- and right-sided diaphragmatic injuries occur at similar frequencies, suggesting that right- injuries are underdiagnosed [8,9].

In the present study, the frequencies of left- and right-sided diaphragmatic injuries were 54.7% and 43.4%, respectively. Interestingly, only four cases of right-sided injury were diagnosed intraoperatively, despite preoperative CT evidence of hepatic herniation. This suggests that the true incidence of right-sided injuries may be underestimated either because surgery was not performed or because the injury was missed on CT. Although small lacerations without obvious injuries can also be missed on imaging on the left side, the diagnostic challenge is generally greater on the right side. Furthermore, some patients who died during resuscitation after arrival at the emergency department may have had an undiagnosed right-sided diaphragmatic injury.

### 4.2. Diagnosis of Diaphragmatic Injury

Chest radiography has a low diagnostic yield (approximately 25%) for diaphragmatic injury [10], leading to delayed or missed diagnoses. In contrast, single-slice CT has reported sensitivities for left- and right-sided diaphragmatic injury of 78% and 50%, respectively, whereas multidetector CT achieves specificities of 100% and 83%, respectively [11]. However, without interpretation by an expert radiologist, initial review of these images by emergency physicians or trauma surgeons may fail to identify diaphragmatic injuries [12].

**Figure 2:**
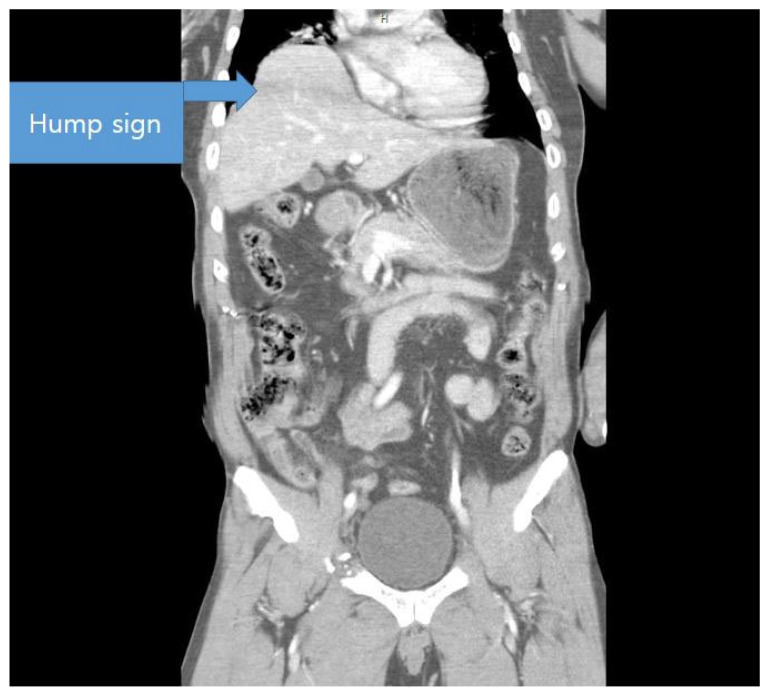
**Hump sign**. Computed tomography image showing partial herniation of the liver into the thoracic cavity, indicating right-sided diaphragmatic injury.

**Figure 3:**
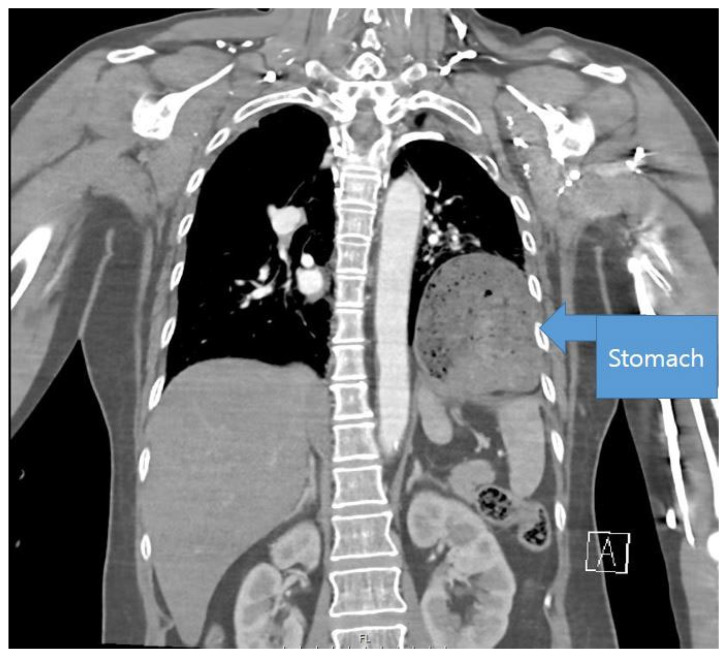
**Stomach herniation**. Computed tomography image showing partial herniation of the stomach into the thoracic cavity, indicating left-sided diaphragmatic injury.

The most definitive finding on CT is herniation of the abdominal organs into the thoracic cavity. In right-sided injuries, part of the liver may herniate (Figure 2), whereas in left-sided injuries, the stomach, spleen, colon, or adipose tissue may be displaced into the thoracic cavity (Figure 3). These findings can facilitate the diagnosis of diaphragmatic injury [13,14]; however, certain conditions, such as congenital hernia or diaphragmatic eventration, can lead to false-positive diagnoses [15].

Partial tears in the diaphragm are particularly difficult to detect, even with CT, and unstable patients may not tolerate CT. In such cases, laparoscopy has proven valuable for the diagnosis and treatment of patients with suspected blunt abdominal trauma who are hemodynamically stable [16,17]. When CT reveals no intra-abdominal injury requiring laparotomy, video-assisted thoracic surgery may be useful for diagnosis and treatment [11].

While penetrating injuries with a trajectory clearly impacting the diaphragm may raise suspicion of diaphragmatic involvement, blunt trauma rarely allows such prediction. In this study, despite initial CT imaging, diaphragmatic injuries were diagnosed in only 51% of patients preoperatively. The preoperative recognition rates were 69% and 30.4% for left-sided and right-sided injuries, respectively, indicating a greater preoperative diagnostic challenge for right-sided injuries. Therefore, to avoid missed diagnoses, the injury mechanism must be considered, physical examinations and imaging should be repeated, and surgical intervention should be considered when clinical suspicion remains high [18].

### 4.3. Mechanisms and Factors Associated with Diaphragmatic Injury

Pazooki et al. reported that blunt trauma to the lower chest can cause diaphragmatic injury due to rib fractures and that splenic injury, lung injury, and hepatic bleeding may indicate diaphragmatic injury [19]. In the present study, among patients whose diaphragmatic injury was surgically confirmed, lower rib fractures were observed in 79.2% (42/53), and hemothorax was identified in 96.2% (51/53). In addition, blunt diaphragmatic injury has been associated with motor vehicle collisions, with hemothorax being the most relevant factor [20].

In several cases in which diaphragmatic injury was not diagnosed preoperatively, exploratory thoracotomy was performed because of persistent hemothorax or worsening chest radiographic findings which revealed partial tears of the diaphragm caused by fractured rib segments. Hemothorax can result from ongoing bleeding from the injuried diaphragm into the pleural cavity, or from rib fractures and lung injuries. In this study, CT confirmed hemothorax in the vast majority of cases (96.2%). Diaphragmatic injury may occur either when sharp rib fragments tear the muscle or there is increased intra-abdominal pressure [11,21]. Because small tears, without herniation, are difficult to detect and preoperative identification rates are reported to be 40–50% for left-sided injuries, but only 0–10% for right-sided injuries, surgical exploration should be considered when clinical suspicion remains high despite negative CT findings [22]. In our study, nearly half (49%, 26/53) of all surgically confirmed injuries had been undiagnosed preoperatively, suggesting the presence of a substantial number of additional undetected cases. Furthermore, even when CT demonstrated herniation, 7.5% (4/53) of cases were not diagnosed until surgery; all these were right-sided injuries associated with hemothorax, indicating that underdiagnosis remains a concern.

In this study, we sought to identify clinical factors that can raise suspicion of diaphragmatic injuries during the initial evaluation of trauma patients. Traffic accidents were the most common cause of such injuries. Diaphragmatic injuries were not identified preoperatively, despite imaging, in 49.1% of cases. Undiagnosed patients more often had pneumothorax, lower rib fractures, and liver injuries. Left-sided injuries were more often diagnosed preoperatively than right-sided injuries, and more frequently involved traffic accidents and abdominal organ herniation into the thoracic cavity. When definite herniation into the thorax could not be identified, pneumothorax strongly indicated possible diaphragmatic injury.

### 4.4. Limitations

In this study, rib fractures were common. In some cases, the fractured rib fragments caused partial tears of the diaphragm, occasionally penetrating the liver. Because thoracotomy is generally performed only in cases of persistent hemothorax or flail chest, it is likely that undetected diaphragmatic injuries exist in non-operated cases with lower rib fractures.

Given the relatively low incidence of diaphragmatic injury, the sample size was small, limiting the statistical power of the study. Furthermore, the retrospective design of the study impacts the strength of our conclusions. Retrospective studies typically rely on past data, which can lead to incomplete, inaccurate, or biased information. Furthermore, single-center studies often have limited study subjects, making it difficult to generalize the results and increasing the risk of bias. Single-center studies, in particular, often reflect the specific characteristics of a specific institution, making them difficult to apply to other institutions or the general population. Specifically, due to the geographical characteristics of the location of this organization, Gangwon-do has an aging population, which limits its ability to represent the metropolitan area and all regions of Republic of Korea.

Therefore, prospective, multicenter studies are needed to obtain more reliable data, expand the generalizability and applicability of the study results, and confirm the effectiveness in various patient groups and settings.

## 5. Conclusions

In patients with multiple, severe traumatic injuries, even if CT does not reveal a diaphragmatic injury, the possibility of occult injury should be considered when a high ISS accompanies hemoperitoneum, hemothorax, lower rib fractures, or extremity injuries. It should be noted that pneumothorax has been identified as the most likely factor to suspect diaphragmatic damage, especially in cases where herniation, which can be easily confirmed radiologically, is absent.

In particular, the mechanism of injury should be considered and, if appropriate, clinicians should maintain a high index of suspicion and should perform repeated physical examinations and imaging assessments. If diaphragmatic injury is strongly suspected, surgical intervention should be considered to confirm the diagnosis.

## Figures and Tables

**Table 1:** General characteristics of blunt diaphragm injury patients.

Variable	Value
Total number of patients	**53**
Sex	
Male	42 (79.2%)
Female	11 (20.8%)
Age (years), Mean ± SD	56.4 ± 15.7
Age ≥ 65 years	14 (26.4%)
Mechanism of Injury	
Traffic accident	34 (64.2%)
Fall	12 (22.6%)
Other	7 (13.2%)
Diaphragm injury location	
Left	29 (54.7%)
Right	23 (43.4%)
Bilateral	1 (1.9%)
Associated injury	
Herniation	24 (45.3%)
Hemothorax	51 (96.2%)
Pneumothorax	31 (58.5%)
Hemoperitoneum	42 (79.2%)
Liver injury	25 (47.2%)
Spleen injury	8 (15.1%)
Bowel injury	3 (5.7%)
Preoperative identification	27 (50.9%)
Time to surgery (min), Median (IQR)	176 (88–2599)
Delay > 1 day	18 (34.0%)
Head and neck AIS score ≥ 2	15 (28.3)
≥3	4 (7.6)
Face AIS score ≥ 2	4 (7.6)
≥3	0
Thorax AIS score ≥ 2	0
≥3	53 (100)
Abdomen AIS score ≥ 2	28 (52.8)
≥3	13 (24.5)
Extremities AIS score ≥ 2	25 (47.2)
≥3	13 (24.5)
ISS, Median (IQR)	24 (16–29)

SD: standard deviation, AIS: Abbreviated Injury Scale, ISS, Injury Severity Score; IQR: interquartile range.

**Table 2:** Comparison of groups according to severity.

Variable	Non-Severe (*n* = 12)		Severe (*n* = 41)		Total (*n* = 53)		*p*-Value
	*n*	%	*n*	%	*n*	%	
Sex							
Male	10	83.3	32	78.0	42	79.2	1
Female	2	16.7	9	22.0	11	20.8	
Age (years), Mean ± SD	61.3 ± 7.8		55.0 ± 17.2		56.4 ± 15.7		0.0813
Age ≥ 65 years	3	25.0	11	26.8	14	26.4	1
Mechanism of injury							
Traffic accident	2	16.7	32	78.0	34	64.2	<0.0001
Fall	5	41.7	7	17.1	12	22.6	
Other	5	41.7	2	4.9	7	13.2	
Injury location							
Left	5	41.7	24	58.5	29	54.7	0.4809
Right	7	58.3	16	39.0	23	43.4	
Bilateral	0	0.0	1	2.4	1	1.9	
Associated injury							
Herniation	1	8.3	23	56.1	24	45.3	0.0035
Hemothorax	12	100.0	39	95.1	51	96.2	1
Pneumothorax	9	75.0	22	53.7	31	58.5	0.3182
Rib fracture	12	100.0	30	73.2	42	79.2	0.0517
Hemoperitoneum	0	0.0	25	61.0	25	47.2	0.0001
Liver injury	2	16.7	6	14.6	8	15.1	1
Spleen injury	0	0.0	8	19.5	8	15.1	0.1748
Bowel injury	0	0.0	3	7.3	3	5.7	1
Preoperative identification	3	25.0	24	58.5	27	50.9	0.0410
Time to surgery (min), Median (IQR)	2898 (1441–5097)		110 (85–229)		176 (88–2599)		0.0003
Delay > 1 day	9	75.0	9	22.0	18	34.0	0.0010
AIS ≥ 2 by body region							
Head and neck	1	8.3	14	34.1	15	28.3	0.1437
Face	0	0.0	4	9.8	4	7.6	0.5632
Thorax	12	100	41	100	53	100	
Abdomen	5	41.7	32	78.1	37	69.8	0.0292
Extremities	0	0	28	68.29	28	52.83	<0.0001
AIS ≥ 3 by body region							
Head and neck	0	0.0	4	9.8	4	7.6	0.5632
Face	0	0.0	0	0.0	0	0.0	
Thorax	12	100	41	100	53	100	
Abdomen	0	0.0	13	31.7	13	24.5	0.0256
Extremities	0	0.0	13	31.7	13	24.5	0.0256

SD: standard deviation, AIS: Abbreviated Injury Scale, IQR: interquartile range.

**Table 3:** Comparison of groups according to diaphragmatic injury site.

Variable	Left Side (*n* = 29)		Right Side (*n* = 23)		Total (*n* = 52)		*p*-Value
	*n*	%	*n*	%	*n*	%	
Sex							
Male	22	75.86	20	86.96	42	80.77	0.4815
Female	7	24.14	3	13.04	10	19.23	
Age (years), Mean ± SD	58.24 ± 15.1		52.78 ± 15.23		55.83 ± 15.26		
Age ≥ 65 years	9	31.03	4	17.39	13	25.00	0.2591
Mechanism of injury							
Traffic Accident	21	72.41	12	52.17	33	63.46	0.0174
Fall	4	13.79	8	34.78	12	23.08	
Others	4	13.79	3	13.04	7	13.46	
Preoperative identification	20	68.97	6	26.09	26	50.00	0.0021
Time to surgery (min), Median (IQR)	110 (85–1422)		1420 (136–4200)		181.5 (87–2655.5)		0.0277
Delay > 1 day	7	24.14	11	47.83	18	34.62	0.0745
Associated injury							
Herniation	15	51.72	8	34.78	23	44.23	0.2218
Hemothorax	27	93.10	23	100.00	50	96.15	0.497
Pneumothorax	16	55.17	15	65.22	31	59.62	0.4634
Rib fracture	23	79.31	18	78.26	41	78.85	1
Hemoperitoneum	14	48.28	10	43.48	24	46.15	0.7303
Liver injury	1	3.45	7	30.43	8	15.38	0.0158
Spleen injury	8	27.59	0	0.00	8	15.38	0.0064
Bowel injury	2	6.90	1	4.35	3	5.77	1

SD: standard deviation, AIS: abbreviated injury scale, IQR: interquartile range. This table presents data excluding one patient with bilateral diaphragmatic injury.

**Table 4:** Comparison of groups according to preoperative identification of diaphragm injury.

Variable	Undiagnosed Preoperatively (*n* = 26)		Diagnosed Preoperatively (*n* = 27)		Total (*n* = 53)		*p*-Value
	*n*	%	*n*	%	*n*	%	
Sex							
Male	22	84.62	20	74.07	42	79.25	0.3442
Female	4	15.38	7	25.93	11	20.75	
Age (years), Mean ± SD	55.96 ± 12.16		56.85 ± 18.73		56.42 ± 15.71		0.8377
Age ≥ 65 years	6	23.08	8	29.63	14	26.42	0.5886
Mechanism of injury							
Traffic accident	12	46.15	22	81.48	34	64.15	0.0290
Fall	9	34.62	3	11.11	12	22.64	
Other	5	19.23	2	7.41	7	13.21	
Time to surgery (min), Median (IQR)	1485 (88–4252)		121 (85–222)		176 (88–2599)		0.0434
Delay > 1 day	13	50.00	5	18.52	18	33.96	0.0156
Diaphragm Injury location							
Left	9	34.62	20	74.07	29	54.72	0.0033
Right	17	65.38	6	22.22	23	43.40	
Bilateral	0	0.00	1	3.70	1	1.89	
Associated injury							
Herniation	4	15.38	20	74.07	24	45.28	<0.0001
Hemothorax	25	96.15	26	96.30	51	96.23	0.9783
Pneumothorax	20	76.92	11	40.74	31	58.49	0.0075
Rib fracture	23	88.46	18	66.67	41	77.36	0.0214
Hemoperitoneum	10	38.46	15	55.56	25	47.17	0.2127
Liver injury	7	26.92	1	3.70	8	15.09	0.0243
Spleen injury	3	11.54	5	18.52	8	15.09	0.7040
Bowel injury	1	3.85	2	7.41	3	5.66	1

SD: standard deviation, AIS: abbreviated injury scale, IQR: interquartile range.

**Table 5:** Comparison of groups according to abdominal organ herniation.

Variable	Non-Herniation (*n* = 29)		Herniation (*n* = 24)		Total (*n* = 53)		*p*-Value
	*n*	%	*n*	%	*n*	%	
Sex							
Male	25	86.21	17	70.83	42	79.25	0.1939
Female	4	13.79	7	29.17	11	20.75	
Age (years), Mean ± SD	54.17 ± 14.97		59.13 ± 16.46		56.42 ± 15.71		0.2571
Age ≥ 65 years	6	20.69	8	33.33	14	26.42	0.2987
Mechanism of injury							
Traffic Accident	14	48.28	20	83.33	34	64.15	0.0321
Fall	9	31.03	3	12.50	12	22.64	
Others	6	20.69	1	4.17	7	13.21	
Preoperative identification	7	24.14	20	83.33	27	50.94	<0.0001
Time to Surgery (min), Mean ± SD	2533 (136–3084)		97 (78.5–209.5)		176 (88–2599)		0.0249
Delay > 1 day	14	48.28	4	16.67	18	33.96	0.0156
Diaphragm Injury location							
Left	14	48.28	15	62.50	29	54.72	0.2152
Right	15	51.72	8	33.33	23	43.40	
Both	0	0.00	1	4.17	1	1.89	
Associated injury							
Hemothorax	28	96.55	23	95.83	51	96.23	1
Pneumothorax	25	86.21	6	25.00	31	58.49	<0.0001
Rib Fracture	27	93.10	15	62.50	42	79.25	0.0145
Hemoperitoneum	10	34.48	15	62.50	25	47.17	0.0420
Liver Injury	5	17.24	3	12.50	8	15.09	0.7153
Spleen Injury	4	13.79	4	16.67	8	15.09	1
Bowel Injury	0	0.00	3	12.50	3	5.66	0.0864
AIS ≥ 2 by body region							
Head and Neck	7	24.1	8	33.3	15	28.3	0.4595
Face	2	6.9	2	8.3	4	7.6	1
Thorax	29	100	24	100	53	100	
Abdomen	18	62.1	19	79.2	37	69.8	0.1771
Extremities	11	37.9	17	70.8	28	52.8	0.0169
AIS ≥ 3 by body region							
Head and Neck	1	3.5	3	12.5	4	7.55	0.3178
Face	0	0.0	0	0.0	0	0.0	
Thorax	29	100	24	100	53	100	
Abdomen	6	20.69	7	29.17	13	24.53	0.4752
Extremities	5	17.2	8	33.3	13	24.5	0.1753

SD: standard deviation, AIS: abbreviated injury scale, IQR: interquartile range.

**Table 6:** Risk factor of diaphragmatic injury in patients with preoperatively unrecognized diaphragmatic injury and in non-herniation cases.

Variable	Odds Ratio (95% Confidence Interval)
No Recognition of Diaphragm Injury before operation	
Right Diaphragm Injury	10.832 (1.987–59.052)
Herniation to thoracic cavity	0.052 (0.010–0.280)
Herniation to thoracic cavity	
Pneumothorax	53.759 (6.128–471.635)
AIS score ≥ 2 for extremity injury	0.081 (0.009–0.707)

AIS: abbreviated injury scale.

## Data Availability

The datasets used and/or analyzed in the current study are available from the corresponding author upon reasonable request.

## Abbreviations

The following abbreviations are used in this manuscript:

CT	Computed tomography
ISS	Injury Severity Score
AP	Anteroposterior
AIS	Abbreviated Injury Scale
OR	Odds ratio

## References

[1] Fair K.A., Gordon N.T., Barbosa R.R., Rowell S.E., Watters J.M., Schreiber M.A. (2015). Traumatic diaphragmatic injury in the American College of Surgeons National Trauma Data Bank: A new examination of a rare diagnosis. Am. J. Surg..

[2] Mizobuchi T., Iwai N., Kohno H., Okada N., Yoshioka T., Ebana H. (2009). Delayed diagnosis of traumatic diaphragmatic rupture. Gen. Thorac. Cardiovasc. Surg..

[3] Pace M., Vallati D., Belloni E., Cavallini M., Ibrahim M., Rendina E.A., Nigri G. (2021). Blunt trauma associated with bilateral diaphragmatic rupture: A case report. Front. Surg..

[4] Bonatti M., Lombardo F., Vezzali N., Zamboni G.A., Bonatti G. (2016). Blunt diaphragmatic lesions: Imaging findings and pitfalls. World J. Radiol..

[5] Mahamid A., Peleg K., Givon A., Alfici R., Olsha O., Ashkenazi I., Israeli Trauma Group (2017). Blunt traumatic diaphragmatic injury: A diagnostic enigma with potential surgical pitfalls. Am. J. Emerg. Med..

[6] Hwang S.W., Kim H.Y., Byun J.H. (2011). Management of patients with traumatic rupture of the diaphragm. Korean J. Thorac. Cardiovasc. Surg..

[7] Alharmoodi F., Ghabra S., Alharthi S. (2023). Incidental traumatic right diaphragmatic rupture: A missed case after trauma. J. Trauma. Inj..

[8] Özkan Ö.F., Özkul F., Guner A., Cekic A.B., Cagılcı A. (2014). Isolated left sided diaphragmatic injury due to blunt trauma, images for clinicians. Eur. J. Gen. Med..

[9] Rashid F., Chakrabarty M.M., Singh R., Iftikhar S.Y. (2009). A review on delayed presentation of diaphragmatic rupture. World J. Emerg. Surg..

[10] Shanmuganathan K., Killeen K., Mirvis S.E., White C.S. (2000). Imaging of diaphragmatic injuries. J. Thorac. Imaging.

[11] Furák J., Athanassiadi K. (2019). Diaphragm and transdiaphragmatic injuries. J. Thorac. Dis..

[12] Chughtai T., Ali S., Sharkey P., Lins M., Rizoli S. (2009). Update on managing diaphragmatic rupture in blunt trauma: A review of 208 consecutive cases. Can. J. Surg..

[13] Desir A., Ghaye B. (2012). CT of blunt diaphragmatic rupture. RadioGraphics.

[14] Rees O., Mirvis S.E., Shanmuganathan K. (2005). Multidetector-row CT of right hemidiaphragmatic rupture caused by blunt trauma: A review of 12 cases. Clin. Radiol..

[15] Bergin D., Ennis R., Keogh C., Fenlon H.M., Murray J.G. (2001). The “dependent viscera” sign in CT diagnosis of blunt traumatic diaphragmatic rupture. AJR Am. J. Roentgenol..

[16] Lin H.F., Chen Y.D., Chen S.C. (2018). Value of diagnostic and therapeutic laparoscopy for patients with blunt abdominal trauma: A 10-year medical center experience. PLoS ONE.

[17] McDonald A.A., Robinson B.R.H., Alarcon L., Bosarge P.L., Dorion H., Haut E.R., Juern J., Madbak F., Reddy S., Weiss P. (2018). Evaluation and management of traumatic diaphragmatic injuries: A Practice Management Guideline from the Eastern Association for the Surgery of Trauma. J. Trauma. Acute Care Surg..

[18] Tavakoli H., Rezaei J., Miratashi Yazdi S.A., Abbasi M. (2019). Traumatic right hemi-diaphragmatic injury: Delayed diagnosis. Surg. Case Rep..

[19] Lundgren J., Mousavie S.H., Negahi A.R., Varga R.N.G., Akyürek L.M., Nakhaei B., Granehed H., Hosseini M., Pazooki D. (2017). Traumatic diaphragmatic ruptures: A 10-year retrospective study. Trauma. Emerg. Care.

[20] Kuo I.M., Liao C.H., Hsin M.C., Kang S.C., Wang S.Y., Ooyang C.H., Fang J.F. (2012). Blunt diaphragmatic rupture—A rare but challenging entity in thoracoabdominal trauma. Am. J. Emerg. Med..

[21] Mahmoud A.F., Eldin Raeia M.M., Abo Elmakarem M.A. (2017). Rupture diaphragm: Early diagnosis and management. J. Egypt. Soc. Cardio Thorac. Surg..

[22] Sharma B., Kafaru M., Agriantonis G., Davis A., Bhatia N.D., Twelker K., Shafaee Z., Dave J., Mestre J., Whittington J. (2025). A case series focusing on blunt traumatic diaphragm injury at a Level 1 trauma center. Biomedicines.

